# The Role of Temporal Envelope and Fine Structure in Mandarin Lexical Tone Perception in Auditory Neuropathy Spectrum Disorder

**DOI:** 10.1371/journal.pone.0129710

**Published:** 2015-06-08

**Authors:** Shuo Wang, Ruijuan Dong, Dongxin Liu, Yuan Wang, Bo Liu, Luo Zhang, Li Xu

**Affiliations:** 1 Otolaryngology—Head & Neck Surgery, Beijing Tongren Hospital, Beijing Institute of Otolaryngology, Capital Medical University, Beijing, China; 2 School of Rehabilitation and Communication Sciences, Ohio University, Athens, Ohio, United States of America; Sun Yat-sen University, CHINA

## Abstract

Temporal information in a signal can be partitioned into temporal envelope (E) and fine structure (FS). Fine structure is important for lexical tone perception for normal-hearing (NH) listeners, and listeners with sensorineural hearing loss (SNHL) have an impaired ability to use FS in lexical tone perception due to the reduced frequency resolution. The present study was aimed to assess which of the acoustic aspects (E or FS) played a more important role in lexical tone perception in subjects with auditory neuropathy spectrum disorder (ANSD) and to determine whether it was the deficit in temporal resolution or frequency resolution that might lead to more detrimental effects on FS processing in pitch perception. Fifty-eight native Mandarin Chinese-speaking subjects (27 with ANSD, 16 with SNHL, and 15 with NH) were assessed for (1) their ability to recognize lexical tones using acoustic E or FS cues with the “auditory chimera” technique, (2) temporal resolution as measured with temporal gap detection (TGD) threshold, and (3) frequency resolution as measured with the Q_10dB_ values of the psychophysical tuning curves. Overall, 26.5%, 60.2%, and 92.1% of lexical tone responses were consistent with FS cues for tone perception for listeners with ANSD, SNHL, and NH, respectively. The mean TGD threshold was significantly higher for listeners with ANSD (11.9 ms) than for SNHL (4.0 ms; *p* < 0.001) and NH (3.9 ms; *p* < 0.001) listeners, with no significant difference between SNHL and NH listeners. In contrast, the mean Q_10dB_ for listeners with SNHL (1.8±0.4) was significantly lower than that for ANSD (3.5±1.0; *p *< 0.001) and NH (3.4±0.9; *p *< 0.001) listeners, with no significant difference between ANSD and NH listeners. These results suggest that reduced temporal resolution, as opposed to reduced frequency selectivity, in ANSD subjects leads to greater degradation of FS processing for pitch perception.

## Introduction

Temporal information in a signal can be partitioned into temporal envelope (E) and fine structure (FS), based on the Hilbert transform, with E defined as amplitude contour of the signal and FS defined as the instantaneous phase information in the signals related to harmonic resolvability [1]. Smith et al. [2] constructed a set of acoustic stimuli, called “auditory chimera”, each having the envelope of one sound and the fine structure of another. This technique provides a way to study the relative importance of E and FS in speech and pitch perception. Perceptual studies have demonstrated that E is sufficient for speech perception in quiet conditions, while FS is important for pitch perception and lexical tone perception [2–3] and perhaps for speech perception in noisy conditions [4–6].

Mandarin Chinese is a tone language with four phonologically distinctive tones, characterized by syllable-level fundamental frequency (F0) contour patterns. These pitch contours are described as high-level (tone 1), rising (tone 2), falling-rising (tone 3), and falling (tone 4) [7]. Wang et al. used the “auditory chimera” technique to demonstrate that as hearing loss of listeners with sensorineural hearing loss (SNHL) becomes more severe, lexical tone recognition relies increasingly on E rather than FS cues, indicating that a degradation of the ability to process FS cues as a function of hearing impairment [8]. Consistent with previous studies, listeners with SNHL have an impaired ability to use FS information in speech or pitch perception [9–12], while their ability to use E cues is equivalent to that in normal-hearing listeners [11,13–14]. Frequency selectivity in listeners with SNHL is reduced due to increased bandwidths of the auditory filters as the hearing impairment becomes more severe. It is possible that reduced frequency selectivity may underlie the impaired ability to process FS information in aforementioned studies [15].

Auditory neuropathy spectrum disorder (ANSD) is an auditory disorder characterized by dys-synchrony of the auditory nerve firing but normal cochlear amplification function. Clinically, it is diagnosed by the presence of otoacoustic emissions (OAEs) and/or cochlear microphonics (CMs) in combination with absent or severely abnormal auditory brainstem responses (ABRs) [16–17]. It is estimated that the prevalence of ANSD ranges between 0.54 and 11% of the hearing-impaired population [18]. Several studies have demonstrated that subjects with ANSD have a dramatically impaired ability for processing temporal information as well as great difficulty in speech perception [19–24], while Vinay and Moore (2007) have suggested that the frequency selectivity in listeners with ANSD may be close to normal [25].

Since listeners with SNHL might have reduced frequency resolution in speech or pitch perception [8, 11–12], but probably normal temporal resolution [26, 13–14], in contrast to listeners with ANSD. It is hypothesized that the ability to process both E and FS information is even more distorted for listeners with ANSD, and that listeners with ANSD may have even more degraded ability to perceive lexical tone than listeners with SNHL. Furthermore, it is predicted that poor temporal resolution rather than the frequency resolution exerts the major detrimental effects on FS cue processing for pitch perception.

In the present study, the “auditory chimera” technique, which was developed to investigate the relative contributions of E and FS cues to Mandarin tone recognition, was used to assess how listeners with SNHL and ANSD achieved lexical tone recognition using either the E or the FS cues. The temporal and frequency resolution was also further assessed in these two groups of listeners.

## Materials and Methods

Three experiments were conducted in the present study. In Experiment I, the temporal resolution of the auditory system was evaluated for three groups of subjects (i.e., normal-hearing (NH) listeners, listeners with SNHL and listeners with ANSD by testing the temporal gap detection (TGD) threshold for each subject. In Experiment II, the frequency selectivity of the auditory system was assessed for each group of subjects by measuring the psychophysical tuning curves (PTCs) for each subject. In Experiment III, chimeric tone tokens developed using the “auditory chimera” technique were employed to examine the relative weights on the acoustic cues (i.e., E or FS) for lexical tone perception for each group of listeners [2–3, 8].

### Subjects

Fifty-eight native Mandarin Chinese-speaking subjects,15 NH subjects (8 females and 7 males), 16 patients with SNHL (10 females and 6 males), and 27 patients with ANSD (9 females and 18 males), were recruited to participate in the study from the Clinical Audiology Center of Beijing Tongren Hospital, China.

**Fig 1:**
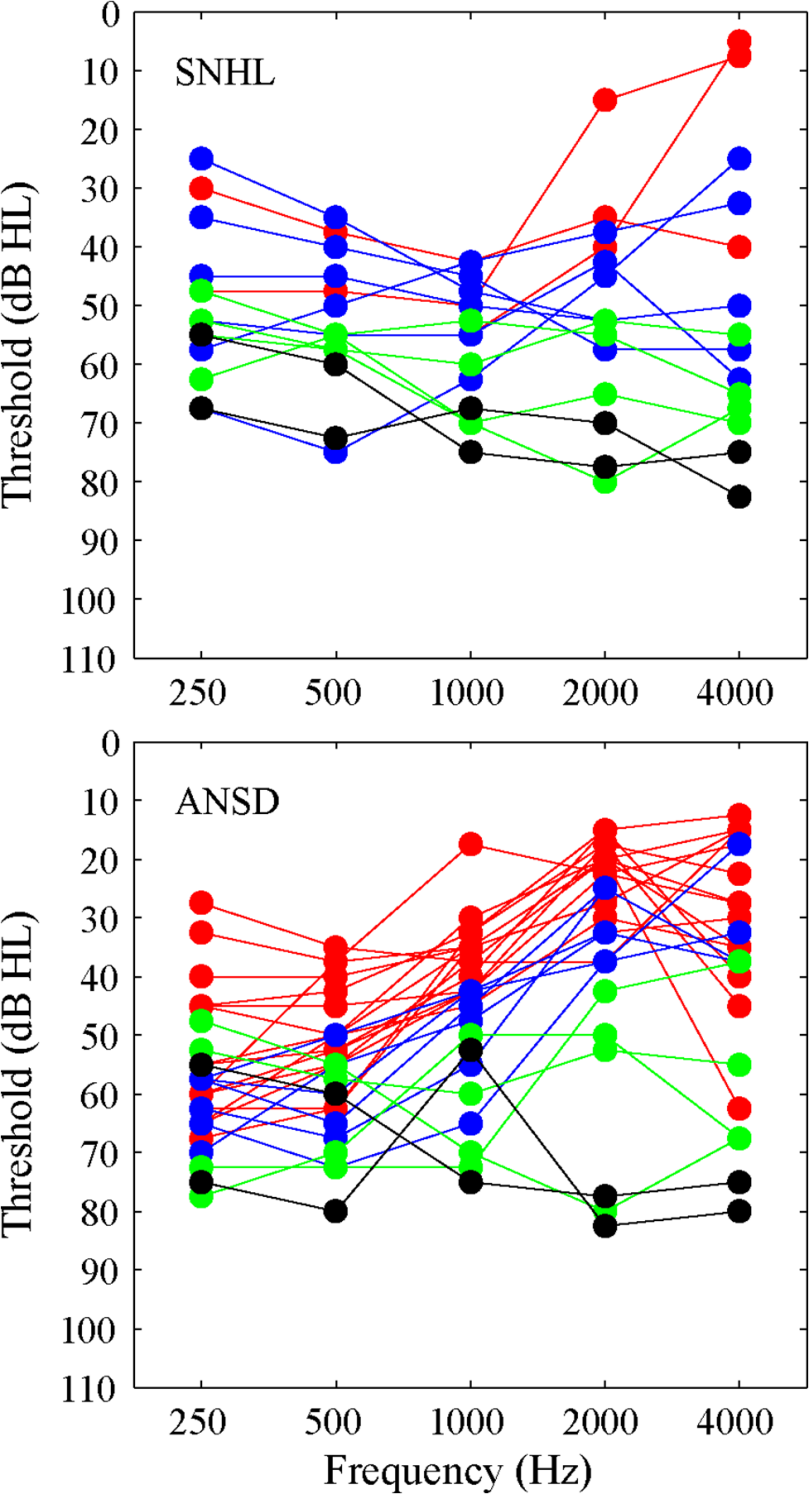
Pure tone hearing threshold for listeners with SNHL (top panel) and with ANSD (bottom panel). Each line represents the averaged hearing threshold for both ears of a subject. Based on the PTA_0.5 to 4 kHz_, red line represents mild hearing loss (26–40 dB HL); blue line represents moderate hearing loss (41–55 dB HL); green line represents moderate to severe hearing loss (56–70 dB HL); and black line represents severe hearing loss (70–90 dB HL).

The NH subjects were aged from 23 to 34 years old (Mean = 26.1, SD = 2.5) and had hearing threshold levels ≤15 dB HL at each octave frequency from 0.25 to 8 kHz. Subjects with SNHL were aged from 15 to 45 years old (Mean = 28.7, SD = 10.5) and had relatively symmetric hearing loss in both ears. The SNHL listeners all had acquired hearing loss in both ears, with the duration of hearing loss ranging from 1.5 to 29 years. Based on the average pure-tone hearing threshold at frequencies of 0.5, 1, 2, and 4 kHz (PTA_0.5 to 4 kHz_), the degree of hearing loss ranged from moderate to severe as shown in the top panel of Fig 1. They all had absent distortion-product OAEs (DPOAEs) at the frequency range of F2 from 0.7 to 6 kHz with a F2/F1 ratio of 1.22. The subjects with ANSD were aged from 18 to 39 years old (Mean = 25.9, SD = 5.2) and most had a “rising” configuration with more hearing loss in low frequencies, as shown in the bottom panel of Fig 1. According to the PTA_0.5 to 4 kHz_, the degree of hearing loss ranged from mild to severe. They all had DPOAEs at the frequency range of F2 from 0.7 to 6 kHz with a F2/F1 ratio of 1.22. The acoustic reflex was absent for pure tone stimuli (105 dB maximal output) at 0.5 to 4 kHz and no auditory brainstem responses were recorded for any of these subjects with the maximum intensity of the click stimuli (103 dB nHL). There was no significant difference in mean age among the NH, SNHL, and ANSD groups (one-way ANOVA, *p* > 0.05).

All subjects gave written informed consent prior to participation in the study protocol, which was reviewed and approved by the Institutional Review Board of Beijing Tongren Hospital. For two subjects who were under 18 years old, the consent form was also signed by the parents.

### Auditory Tests

Temporal gap detection (TGD) threshold in Experiment I was assessed using a TGD program developed by Zeng and colleagues [23]. Briefly, the test stimuli were generated using a broadband (from 20 to 14,000 Hz) white noise, of 500-ms duration with 2.5-ms cosine-squared ramps, and a silent gap was produced in the centre of the target noise. Two uninterrupted reference noises were also generated, and a three-alternative, forced-choice procedure was used to measure the TGD thresholds.

Psychophysical tuning curves (PTCs) of the auditory system were measured in Experiment II using a fast method (Sweeping PTC, SWPTC), developed by Sek and Moore [27]. PTCs were tested separately in each ear at 500 and 1000 Hz, with the subjects being required to detect a sinusoidal signal that was pulsed on and off repeatedly in the presence of a continuous noise masker. The sinusoidal signals were fixed at 500 Hz and 1000 Hz, respectively, and presented at 15 dB sensation level (SL). The masker was a narrowband noise, slowly swept in frequency. For instance, to mask the signal at 1000 Hz, the lowest frequency of the masker was set at 500 Hz and the highest frequency at 1500 Hz. The noise was applied for 240 s, at a bandwidth of 200 Hz, with the rate of change of the masker level set at 1 dB/s. The initial noise level was set to 40 dB below the signal level. The frequency at the tip of the PTC was estimated using a four-point moving average method (4-PMA), and the Q_10dB_ (i.e., signal frequency divided by the PTC bandwidth at the point 10 dB above the minimum level, *f*_sin_/bandwidth) value was used to assess the sharpness of the PTC [27], with a greater Q_10dB_ value reflecting a sharper PTC.

For Experiment III, 10 sets of Chinese monosyllables (including “bai”, “di”, “tan”, “fei”, “guo”, “hu”, “liu”, “ma”, “qü”, and “she”), with four tone patterns each, were used to generate a total of 40 commonly used Chinese words. These words were recorded in an acoustically treated booth from an adult male and an adult female native Mandarin speaker whose fundamental frequency (F0) was around 180 Hz for the male and around 300 Hz for the female. In order to eliminate the effect of duration cues on tone perception, the speakers were asked to record these 40 monosyllabic words multiple times, and the tokens in which the durations of four tones in each monosyllabic word were within 5-ms precision were chosen as the original tone tokens. Speech signals were captured through an M-Audio Delta 64 PCI digital recording interface connected to a computer. Recordings were made at a 44.1-kHz sampling rate and 16-bit quantization. Thus, a total of 80 tone tokens were recorded digitally.

Chimeric tone tokens were created using the “auditory chimera” technique in this study [2–3, 8]. The chimeric tokens were generated in a condition with 16 channels. These FIR band-pass filters with nearly rectangular response were equally spaced on a cochlear frequency map [28]. The overall frequency range for chimera synthesis was between 80 and 8820 Hz. The transition over which adjacent filters overlap was 25% of the bandwidth of the narrowest filter. One modification from previous studies [8] was that a lowpass filter (cut-off at 64 Hz) for extraction of the envelopes was adopted in order to avoid the blurriness between E and FS in the chimeric stimuli. For instance, two tokens of the same syllable but with different tone patterns (e.g., “ma” with tone 1 and “ma” with tone 2) were passed through 16 band-pass filters to split each sound into 16 channels. The output of each filter was then divided into its E and FS using a Hilbert transform. Then, the E of the output in each filter band was exchanged with the E in that band for the other token to produce the single-band chimeric wave. The single-band chimeric waves were summed across all channels to generate two chimeric stimuli (e.g., one with E of “ma tone 1” and FS of “ma tone 2”, the other with E of “ma tone 2” and FS of “ma tone 1”). With 4 different tones in Mandarin, a total of 12 chimeric tone tokens were generated for each set of monosyllables, providing a total of 240 chimeric tone tokens (i.e., 12 chimeric combinations × 10 sets of monosyllables × 2 voices). Overall, in combination with the 80 original unprocessed tone tokens, a total of 320 tokens were used in the tone test, employing a four-alternative, forced-choice procedure.

### Procedure

All experiments were conducted in an acoustically treated booth. Subjects were instructed on the procedures to be employed and undertook practice sessions to familiarize them with the test for each experiment. Listeners with ANSD typically took 15–20 mins in the practice session whereas listeners with NH and SNHL took about 5–10 mins in the practice sessions. On average, the experiments lasted for approximately 10–15 mins (Experiment I), 30–40 mins (Experiment II), and 20–25 mins (Experiment III) for each subject, respectively, and subjects were allowed to take breaks between each experiment.

In Experiment I, the TGD threshold of left and right ear was measured separately for each subject using a three-alternative, forced-choice procedure. The stimuli were presented unilaterally via a MADSEN TDH-50P headphone. The intensity level of the white noise was set at the most comfortable loudness level for all subjects. Subjects were required to use a computer mouse to select the noise token that had a silent interval in the middle. A two-down one-up adaptive tracking procedure was adopted [29], and the TGD threshold at 70.7% correct was automatically calculated by the TGD program.

In Experiment II, the PTC was measured at 500 Hz and 1000 Hz for each ear. The order of the testing ear and target frequency was randomized for each subject, and the stimulus was presented unilaterally through a MADSEN TDH-50P headphone. Prior to the test, the absolute hearing threshold at the target frequency was measured using the SWPTC software to achieve the correct signal presentation level for the test. At the beginning of the test, the sinusoidal signal was presented without the noise masker, and the subject was asked to listen for this signal carefully during the entire task. A 200-Hz-wide noise masker at various center frequencies was then presented in the same ear at a low intensity level, so that the sinusoidal signal was still audible to the subject. The subject was asked to indicate this by pressing and holding down the space bar of the computer as long as the sinusoidal signal was audible. The level of the noise masker was increased at a rate of 1 dB/s, and the subject was instructed to release the space bar once the sinusoidal signal was no longer audible in the presence of the noise. At this point, the level of the noise masker was decreased until the sinusoidal signal was audible again. The PTC was plotted for each target frequency, and the Q_10dB_ value calculated.

For “auditory chimera” test in Experiment III, a GSL-16 clinical audiometer connected to a dedicated computer was used to adjust the intensity of the chimeric tone tokens. A custom graphical user interface (GUI) written in MATLAB, as described previously [8], was used to present the stimuli and to record the responses. The chimeric tone tokens were presented bilaterally through MADSEN TDH-50P headphones in random orders, with the stimuli set at the most comfortable loudness level for subjects with ANSD and SNHL and fixed at 65 dB SPL for NH subjects. The subjects were required to select which tone (or what Chinese monosyllabic word) was in the chimeric tone token that they had heard, and the percentage of tone responses that were either consistent with E or FS were calculated. The percent-correct scores of tone perception performance using the original, unprocessed tone tokens were also calculated.

### Statistical analysis

The results were analyzed statistically using Statistics Package for Social Science (SPSS) 16.0.

## Results

### Temporal gap detection

**Fig 2:**
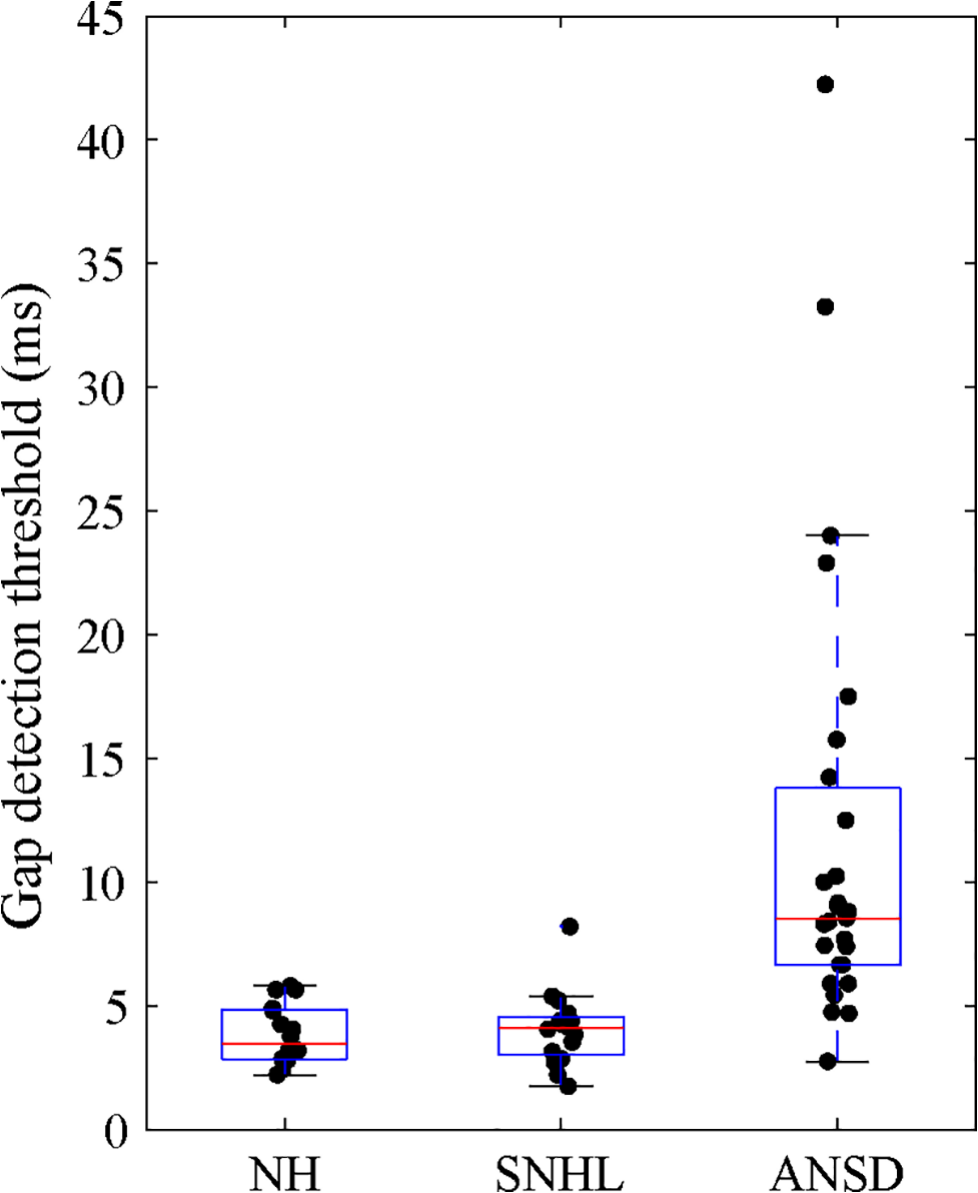
Temporal gap detection thresholds for subjects with NH, with SNHL, and with ANSD. The horizontal lines of the box represent 25th, 50th, and 75th percentiles and the whiskers represent the range of the data. Individual subjects are represented by the solid dot.

The TGD threshold for three groups of subjects is shown in Fig 2. Welch’s test in a one-way ANOVA was found that significant difference of TGD thresholds among the three groups of subjects [F(2,56) = 9.9, *p* < 0.001]. Post hoc Tamhane’ T2 correction test analysis indicated that the mean TGD thresholds for both ears was significantly higher in listeners with ANSD (11.9ms) than in SNHL (4.0ms; *p* < 0.001) and in NH (3.9ms; *p* < 0.001) listeners, with no significant difference between SNHL and NH listeners. Unlike NH and SNHL subjects, the TGD in subjects with ANSD was highly variable (range = 2.7 to 42.3ms), with only 4 of the 27 listeners with ANSD demonstrating TGD thresholds within the normal limits (2.7 to 6 ms).

### Psychophysical tuning curves

**Fig 3:**
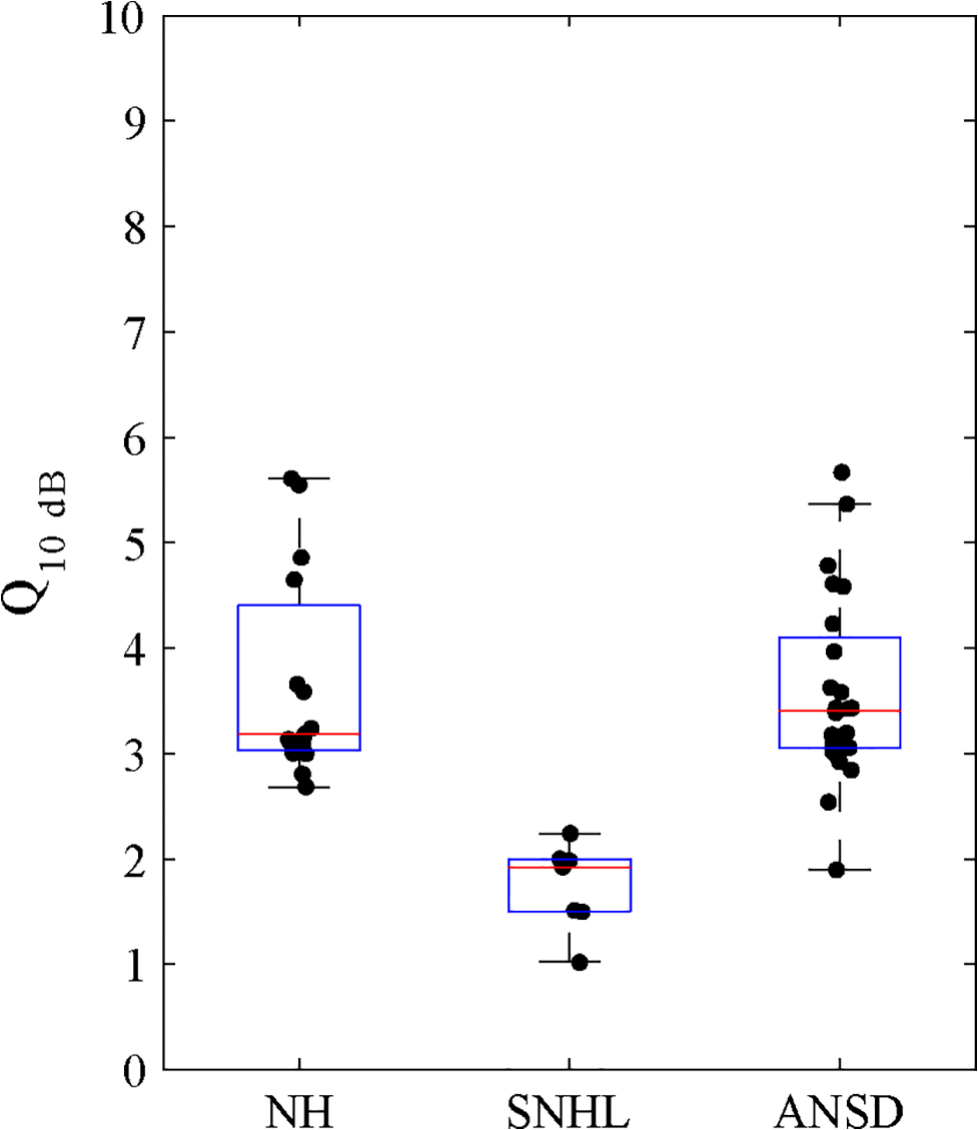
The Q_10dB_ values of psychophysical tuning curves for subjects with NH, with SNHL, and with ANSD. The horizontal lines of the box represent 25th, 50th, and 75th percentiles and the whiskers represent the range of the data. Individual subjects are represented by the solid dot.

Paired-samples t test showed that the Q_10dB_ values of PCTs measured at 500 Hz and 1000 Hz, and in both ears were not significantly different in any groups of subjects. Therefore, the mean values for the two frequencies were used (Fig 3). The values of Q_10dB_ for NH subjects ranged from 2.3 to 6.2 (mean = 3.4, SD = 0.9). The Q_10dB_ values could not be determined in 9 of the 16 SNHL subjects assessed, because of their very broad PCTs. However, the Q_10dB_ values in the remaining 7 subjects ranged from 1.0 to 2.3 (mean = 1.8, SD = 0.4). On the other hand, the Q_10dB_ values could be determined in 24 of the 27 ANSD subjects assessed, and the range was between 1.6 and 7.0 (mean = 3.5, SD = 1.0). Welch’s test in a one-way ANOVA demonstrated a significant difference of Q_10dB_ values among the three groups of subjects [F(2, 125) = 63.7, *p* < 0.001], with post hoc Tamhane’ T2 correction test further showing significant differences between ANSD and SNHL subjects (*p* < 0.001) as well as between with NH and SNHL subjects (*p* < 0.001). The differences between Q_10dB_ values of the NH and ANSD subjects were not significantly different.

### Lexical tone perception

**Fig 4:**
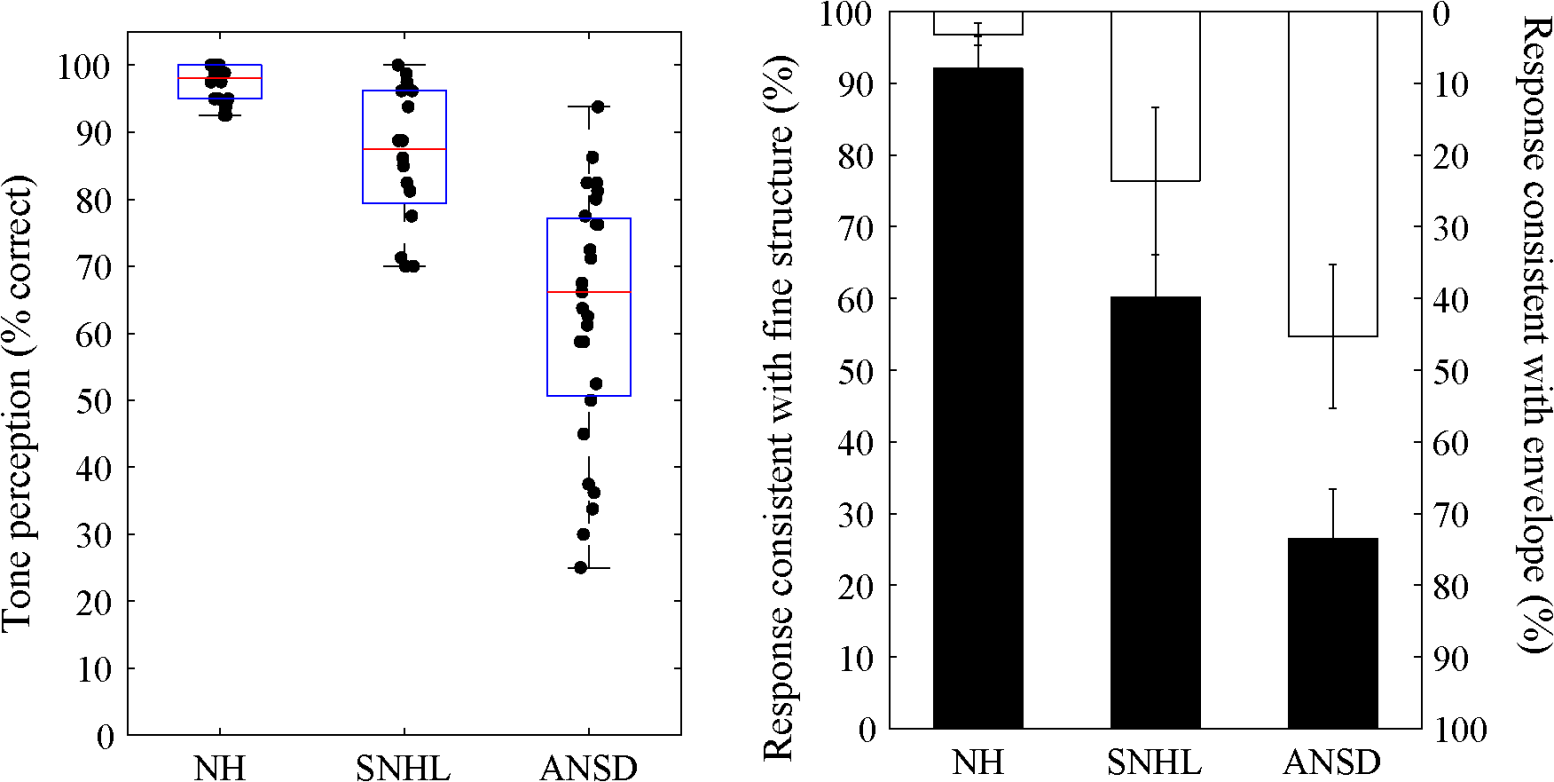
Tone perception performance with the original, unprocessed tone tokens (left) and with chimeric tone tokens (right) for subjects with NH, with SNHL, and with ANSD. Left: The horizontal lines represent 25th, 50th, and 75th percentiles and the whiskers represent the range of the data. Individual subjects are represented by the solid dot. Right: Bars represent mean percentages of the tone responses consistent with FS (filled bar, left ordinate) and E (open bar, right ordinate) for the three groups of subjects. Error bar represents standard deviation.

The accuracy of tone perception to the original tone tokens was 97.2%, 86.5%, and 62.8% correct for the NH, SNHL, and ANSD subjects, respectively (Fig 4, left panel). Rational arcsine transformation was performed on lexical tone recognition scores for the three groups of subjects in order to make the percent-correct scores suitable for ANOVA. A one-way ANOVA followed by post hoc tests with Bonferroni correction showed significant differences in lexical tone perception scores among the three groups of subjects [F(2, 56) = 49.0, *p* < 0.001]. For responses to the chimeric tone tokens, 92.1%, 60.2%, and 26.5% of the tone perception responses were found to be consistent with FS of the chimeric tone tokens for the NH, SNHL, and ANSD subjects, respectively, whereas 3.1%, 23.6%, and 45.3% of the tone responses were consistent with E for the three groups (Fig 4, right panel). Welch’s test in a one-way ANOVA showed that the mean percentages of tone responses that were consistent with either FS or E cues were significantly different from each other among the three groups [FS: F(2, 56) = 694.4, *p* < 0.001; E: F(2, 56) = 294.1, *p* < 0.001]. Subjects with SNHL had reduced ability to use FS in lexical tone perception in comparison with NH subjects. However, in relation to subjects with NH and SNHL, subjects with ANSD showed more severely impaired ability to use FS in lexical tone perception, with only 26.5% of the tone responses based on FS. On the other hand, subjects with SNHL showed more tone responses consistent with E than did the NH subjects, as their ability to use FS in tone perception gradually decreased. Subjects with ANSD mainly relied on E in perceiving tones. They also recorded a high error rate in tone responses (33.3% of the responses that were neither consistent with FS nor with E), compared to error rates of 3.8% and 17.4% in the NH and SNHL subjects, respectively.

**Fig 5:**
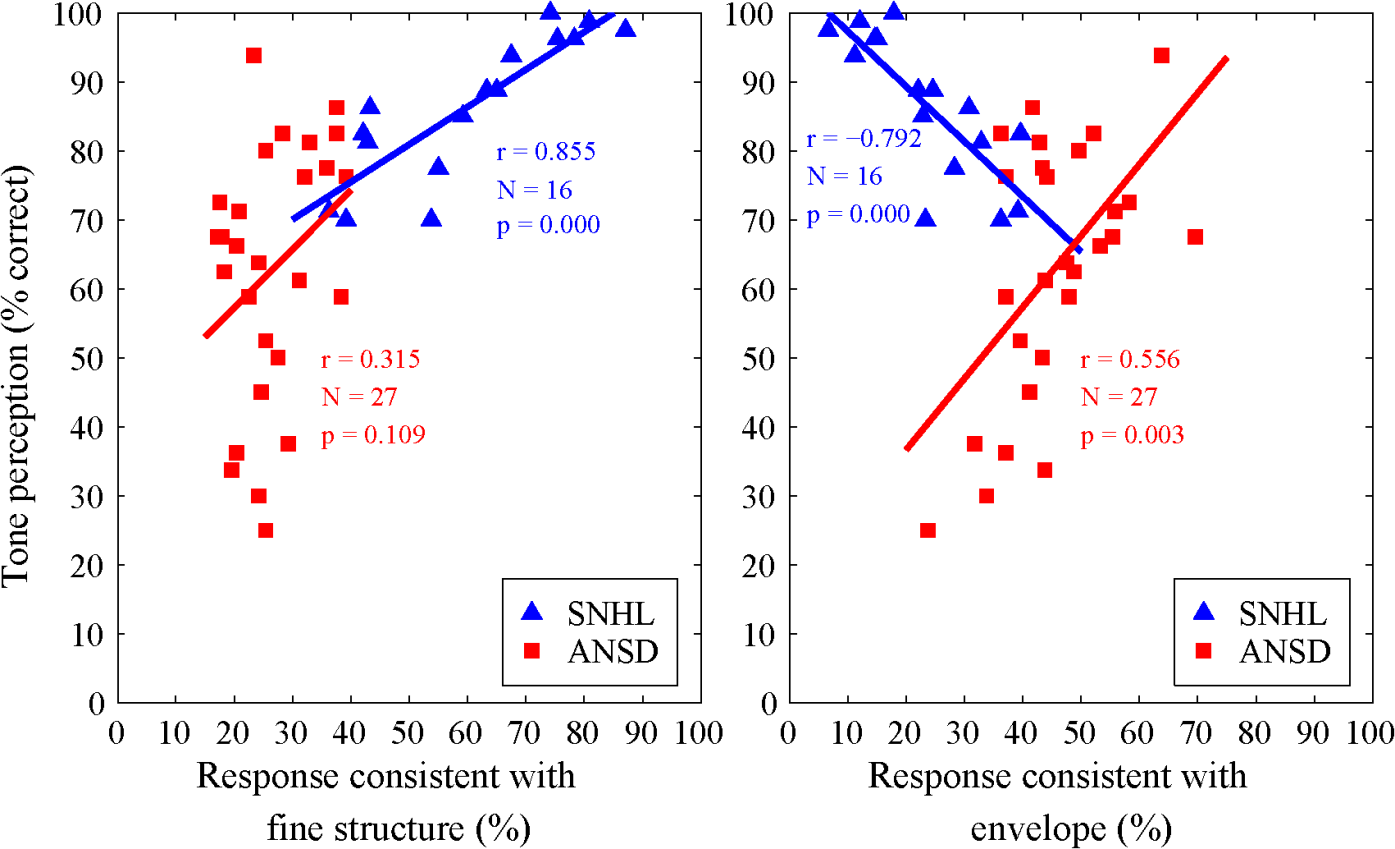
Correlation between tone perception score and tone responses that were consistent with FS (left) and E (right) for subjects with SNHL (triangle) and ANSD (square).

Fig 5 shows the correlation between tone perception score and tone responses that were consistent with FS (left) and E (right) for subjects with SNHL and ANSD. In subjects with SNHL, the tone perception score was significantly correlated with tone responses that were consistent with FS [*r* (16) = 0.855, *p* < 0.001] and significantly negatively correlated with responses consistent with E [*r* (16) = - 0.792, *p* < 0.001]. In contrast, in subjects with ANSD, the tone perception score was significantly correlated with tone responses that were consistent with E [*r* (27) = 0.556, *p* < 0.05], but not with responses consistent with FS.

### Correlation between audiometric thresholds and tone responses for subjects with SNHL and ANSD

**Fig 6:**
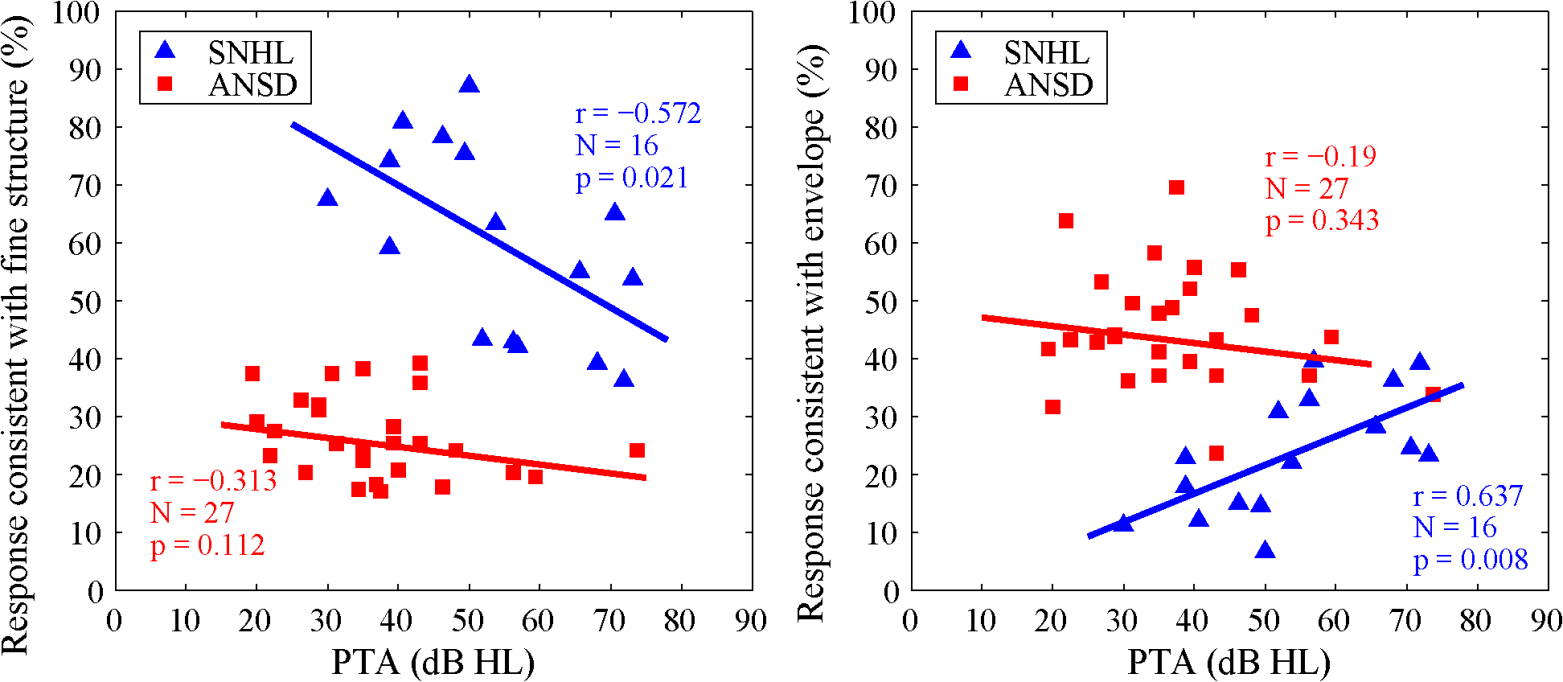
Correlation between the average of pure-tone hearing thresholds (PTA) between 0.5 and 4k Hz and tone responses consistent with fine structure (left) and temporal envelope (right) for both subjects with SNHL (triangle) and ANSD (square).

Fig 6 shows the correlation between the average of PTA_0.5 to 4 kHz_ and tone response performance for SNHL and ANSD subjects. For subjects with SNHL, the tone responses that were consistent with FS were negatively correlated with the PTA_0.5 to 4 kHz_ [*r* (16) = - 0.572, *p* < 0.05] (Fig 6, left panel), whereas the tone responses that were consistent with E were positively correlated with the PTA_0.5 to 4 kHz_ [*r* (16) = 0.637, *p* < 0.05] (Fig 6, right panel). However, for subjects with ANSD, no correlation was observed between the hearing thresholds and tone responses that were consistent with either FS or E [FS: *r* (27) = - 0.313, p = 0.112; E: *r* (27) = - 0.19, p = 0.343].

### Correlation between TGD thresholds and tone perception for subjects with SNHL and ANSD

**Fig 7:**
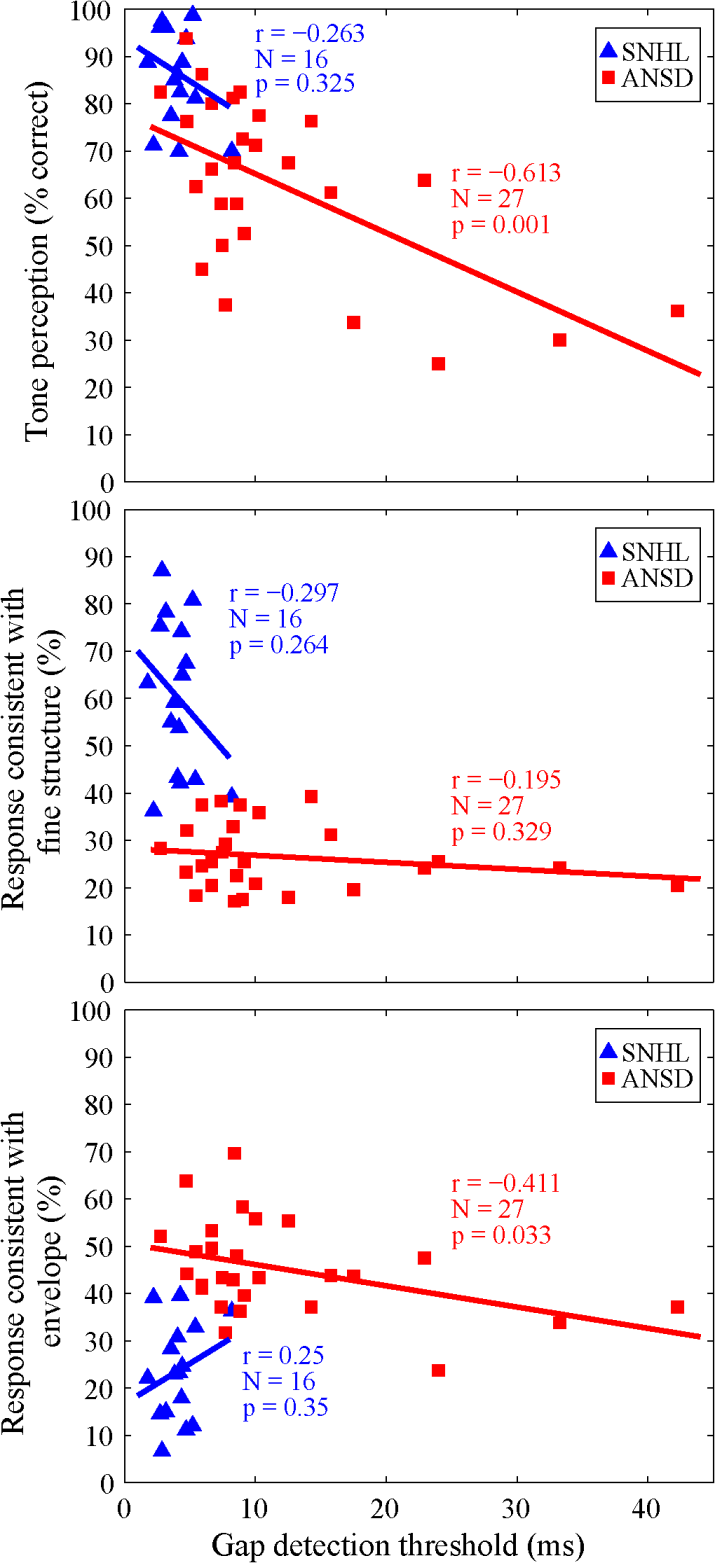
Correlation between TGD threshold and tone perception score (upper), and tone responses consistent with FS (middle) and tone responses consistent with E (lower) across both groups of subjects with SNHL (triangle) and ANSD (square).

The upper panel in Fig 7 shows the correlation between TGD threshold and tone perception score for SNHL and ANSD subjects. The TGD threshold and tone perception score were significantly correlated negatively for subjects with ANSD [*r* (27) = - 0.613, *p* < 0.05]. However, no such correlation was observed in subjects with SNHL. The middle and lower panels of Fig 7 show the correlation between TGD threshold and tone responses consistent with either FS or E in SNHL and ANSD subjects. A significantly negative correlation was observed between TGD threshold and tone responses consistent with E in subjects with ANSD [*r* (27) = - 0.411, *p* < 0.05], but not between TGD threshold and tone responses consistent with FS [*r* (27) = 0.25, *p* > 0.05]. In contrast, there was no correlation between TGD threshold and tone responses consistent with either FS or E in subjects with SNHL.

## Discussion

The present study has demonstrated that listeners with ANSD have deficits in using the FS cues for lexical tone perception, with on average only 26.5% of tone responses being consistent with FS, compared to 60.2% of tone responses being based on FS for listeners with SNHL and 92.1% for listeners with NH. Wang et al. [8] reported that listeners with severe SNHL could achieve 38% of lexical tone responses consistent with FS. However, most listeners with ANSD had mild to moderate hearing loss in the present study, but their ability to use FS for lexical tone perception was found to be even poorer than that of listeners with *severe* SNHL.

There was no correlation between the tone responses that were consistent with FS cues and the audiometric hearing thresholds for listeners with ANSD. This may indicate that ANSD listeners had a severely degraded ability to use FS cues in lexical tone perception no matter which degree of hearing loss they had. Fine structure is important for pitch perception [2, 3], and for speech perception in both steady state and modulating noises [5]. Consistent with the findings from previous studies that listeners with ANSD had extreme difficulties in understanding speech in noise [20, 23–24], it was found that listeners with ANSD also had great difficulties in perceiving lexical tone. Therefore, the deficits in processing FS may partly account for the above-mentioned poor performance in listeners with ANSD.

Moreover, the ability of listeners with ANSD to use E cues for lexical tone perception remained at fairly high levels ranging from 24% to 64%. A positive correlation between the responses consistent with E cues, and the overall lexical tone perception suggests that the relatively good ability to use E cues in listeners with ANSD helped them to achieve tone perception performance at 62.8% correct. However, this level of tone perception performance was much lower compared to that of NH listeners, in whom the FS cues could provide perfect tone perception [3, 8]. The tone perception performance in listeners with ANSD was also lower than that in listeners with SNHL who could use the FS cues for tone perception to some extent. Earlier studies using noise vocoders to investigate Mandarin lexical tone perception have indicated that the contributions of E cues in the absence of detailed spectral information to lexical tone perception are not robust, and that NH listeners can achieve 70% to 80% correct at best [1, 30].

The temporal gap detection test was used to evaluate the temporal resolution for both listeners with SNHL and with ANSD in the present study. Consistent with previous studies [23, 31–32], the TGD thresholds for listeners with SNHL were comparable to those for NH listeners as long as the audibility was compensated. However, a majority of listeners with ANSD had great deficits in the temporal gap detection test. Their TGD thresholds were 3 − 4 times greater than normal range at the comfortable loudness level. This finding suggests that listeners with SNHL have close to normal temporal resolution ability with the appropriate audibility, whereas a majority of listeners with ANSD have deficits in temporal resolution. Notably, four listeners with ANSD in the present study had a TGD threshold in the normal range with the appropriate audibility. The tone perception scores for these individuals ranged from 76.3% to 93.8% correct, which was 1 to 3 SDs higher than the average of 62.8% correct for the group of listeners with ANSD. Two of these four individuals performed chimeric tone test comparable to the performance for listeners with severe SNHL in the study by Wang et al. [8] and achieved 35.0–37.5% of the lexical tone responses that were consistent with FS, and 41.8–44.2% of the lexical tone responses that were consistent with E. This suggests that they might still be able to use some FS information to perceive lexical tones. It is possible that although the other two listeners with ANSD could not use FS to perceive lexical tone (< 25%), their ability to use E cues (58.0–63.2%) may compensate for the deficit in processing FS to some extent.

The psychophysical tuning curves were used to evaluate frequency selectivity for listeners with SNHL and ANSD. The Q_10dB_ value of the PTC for a majority of listeners with ANSD was within ± 1 S.D. of that for listeners with NH, indicating that listeners with ANSD have close-to-normal frequency resolution, possibly due to normal outer hair cell functions in these listeners. In contrast, the Q_10dB_ value could not be estimated for almost half of the listeners with SNHL, and for those in whom it could be measured the value of Q_10dB_ was significantly smaller than that for NH listeners or listeners with ANSD. This finding suggests that listeners with SNHL have poor frequency selectivity, possibly due to outer hair cell damage. The finding from the present study that listeners with SNHL had reduced ability to process FS in lexical tone perception, and were still able to use E cues to compensate for this deficit is in accordance with our previous study of a large group of listeners with SNHL (N = 31) [8].

Correlational analysis revealed no association of duration of hearing loss and tone perception performance and TGD thresholds for listeners with SNHL in the present study. The mechanism underlying the reduced ability to process acoustic FS due to SNHL has been investigated in several studies [15, 33–40]. It has been proposed that reduced compression of the basilar membrane input-output function due to the reduced frequency selectivity may amplify the coding of E in the auditory system, and that this enhanced envelope coding may lead to a “relative” deficit in FS coding. However, it is possible that reduced frequency selectivity may underlie the impaired ability to process FS information in speech and pitch perception. Another possible explanation is that acoustic FS may be coded in the phase locking across auditory nervefibres, so the deficits of FS processing may be also attributed to the impaired temporal coding (i.e., phase-locking) in sensorineural hearing-impaired listeners [11–12, 41]. However, the evidence underlying the FS deficit in hearing impairment continues to be debated, as cochlear damage does not appear to systematically reduce the phase-locking ability within the auditory fibers [42–45].

It is important to understand the effects of frequency resolution and temporal resolution on FS cue processing. The present study demonstrated that both degraded frequency resolution and temporal resolution exerted detrimental effects on FS cues processing for lexical tone perception. While listeners with SNHL had normal temporal resolution but poor frequency resolution, their ability to process FS cues for lexical tone perception was nevertheless only slightly affected. In contrast, a majority of listeners with ANSD had degraded temporal resolution, as shown by marked deficits in the TGD test, and could hardly process FS cues for lexical tone perception. Collectively, consistent with the hypothesis, these findings suggest that the detrimental effects of degraded temporal resolution on FS cue processing are far greater than those of degraded frequency resolution.

The exact pathology of ANSD remains unclear. It has been suggested that lesions in the synapse between spiral ganglion neurons and the inner hair cells and/or neural demyelination of the primary neuron fibres, may lead to a large temporal jitter in spike initiation, which results in desynchronized spike discharges [46]. Another study has suggested that loss of the inner hair cells and/or the auditory neurons may lead to reduced amount of spikes discharge [47]. Zeng et al. [24] hypothesised that the desynchronized spike discharges may lead to a time smeared neural representation of the acoustic stimulus. Based on our finding from the present study that the majority of subjects with ANSD showed degraded ability in the gap detection task, it was speculated that the desynchronized spike discharges may remove the ability of the auditory system to detect the onset or offset of the stimuli. The time smeared neural presentation may further lead to difficulty in coding the FS information, which dramatically impacts lexical tone perception performance, with greater severity of desynchronization leading to greater distortion of temporal information processing.

Choosing appropriate management for patients with ANSD presents a clinical dilemma because hearing aid fitting does not provide enough benefits for them to perceive speech in both quiet and noise conditions [46, 48]. The contemporary signal-processing algorithm designed in hearing aids employs nonlinear amplitude compression, which may reduce the temporal amplitude modulation in speech signal. It appears that reinforcing the E cues in speech signals may be a way to improve tone perception and speech perception performance for listeners with ANSD, at least in quiet conditions. On the other hand, it may not be helpful to strengthen the acoustic FS cues for listeners with ANSD since their ability to process FS is nearly diminished. Cochlear implant efficiently delivers the E information to its users and thus represents another promising rehabilitative option for listeners with ANSD [49], although studies have produced variable results, with some showing marked benefits [50, 51], and others reporting speech perception results poorer than those in listeners with SNHL [19]. Future studies are necessary to elucidate the efficacy of fine structure processing strategies in cochlear implants for patients with ANSD, especially with regard to their tone perception or speech perception in noise.

## Conclusions

The present study examined the temporal resolution using a gap detection task in listeners with NH, with SNHL, and with ANSD. Psychophysical tuning curves were also evaluated in the three groups of subjects as a measure of their frequency resolution. Lexical tone perception was tested in all subjects using the original tone tokens and the 16-channel chimeric tone tokens. The results demonstrated that:

1. Lexical tone perception in listeners with ANSD is fairly poor, much poorer than that in listeners with SNHL;
2. The ability to use FS cues for pitch perception as revealed by the percentage responses that are consistent with FS of the chimeric tone tokens decreases as the severity of SNHL increases in the listeners with SNHL, but is nearly diminished in listeners with ANSD irrespective of the degree of hearing thresholds;
3. The ability to use E cues for pitch perception is retained in both listeners with ANSD and SNHL;
4. Listeners with SNHL have poor frequency resolution as revealed by the reduced Q_10 dB_ values in the psychophysical tuning curves whereas listeners with ANSD have normal frequency resolution;
5. Listeners with ANSD have very poor temporal resolution as revealed by the much increased gap detection thresholds whereas listeners with SNHL have normal temporal resolution;
6. Poor temporal resolution rather than the frequency resolution exerts the major detrimental effects on FS cue processing for pitch perception; and
7. Retaining or enhancing temporal envelope cues in the hearing devices (hearing aids or cochlear implants) might be beneficial for listeners with ANSD.
